# Effects of aerobic treadmill exercise on the bone turnover in obese male mice

**DOI:** 10.3389/fcell.2025.1650496

**Published:** 2025-11-06

**Authors:** Yuxiang Du, Guanghua Liu, Limei Zhang, Haiqi Lin, Bo Gao, Lingli Zhang

**Affiliations:** 1 School of Exercise and Health, Shanghai University of Sport, Shanghai, China; 2 Department of Rehabilitation Medicine, Zhongshan Hospital, Fudan University, Shanghai, China; 3 School of Physical Education and Sports Science, South China Normal University, Guangzhou, China; 4 Institute of Orthopedic Surgery, Xijing Hospital, Fourth Military Medical University, Xi’an, China; 5 College of Athletic Performance, Shanghai University of Sport, Shanghai, China

**Keywords:** treadmill exercise, high-fat diet, obesity, bone mass, bone formation, bone resorption

## Abstract

**Introduction:**

This study aims to investigate the effects of aerobic exercise on the bones of obese male mice induced by a high-fat diet and determine the related mechanisms. Results will provide a reference for exercise-related recommendations in the early adulthood of rodents.

**Methods:**

Sixty male C57BL/6 mice were raised to 5 weeks of age and then stratified randomly by body weight into a normal diet group and a high-fat diet group for a 12-week dietary intervention. After 12 weeks, successfully modeled obese male mice were stratified randomly by body weight into an obese control group and an obese exercise group. Twenty mice from the normal diet group were stratified randomly by body weight into a control group and an exercise group. A 10-week aerobic treadmill exercise intervention was conducted, and the feed administered to each group was not changed.

**Results:**

High-fat diet-induced obesity causes abnormalities in body weight, blood glucose, and lipid metabolism in mice, inhibits bone formation, promotes bone resorption, and leads to decreased bone mass in obese male mice.

**Conclusion:**

These findings are mainly related to the inhibition of the WNT signaling pathway and the dysregulation of adipokines. Aerobic exercise can effectively reduce the body weight of obese male mice and alleviate abnormalities in glucose and lipid metabolism. To some extent, it also alleviates the inhibition effect of obesity on WNT pathway signaling, promotes bone formation, and inhibits bone resorption.

## Introduction

Changes in production and lifestyle have gradually increased the proportion of obese people worldwide, especially in developed regions. Obesity is caused by the imbalance of energy intake and consumption and is mainly characterized by overweight and massive fat accumulation. The World Health Organization (WHO) has set body mass index≥ 30 kg/m^2^ as the standard for judging obesity. About 13% of people suffer from obesity globally. If the recent trend continues, then 57.8% of the of the adult population will be overweight or obese by 2030 (Blüher, 2019; Caballero, 2019). Obesity cannot only greatly increase the risk of chronic metabolic diseases such as diabetes and hypertension but also is closely related to the health status of bones (Apovian, 2016).

Osteoporosis (OP) is characterized by decreased bone mineral density (BMD), low bone mass, and increased risk of fracture. The imbalance of bone homeostasis is the most fundamental cause of OP (Felsenberg and Boonen, 2005). The effect of obesity on bone is complex and could be benign. High body weight acts on bones, and it causes strong mechanical stimulation signals felt by bone cells, such as osteoblasts and osteocytes, and increase bone mass. In addition, the increase in estrogen level in obese people can directly promote the activity of osteoblasts, inhibit the activity of osteoclasts, and protect bones (Rinonapoli et al., 2021). However, scholars found that the positive effect of mechanical load on bone mass due to weight gain could not offset the adverse effect of obesity on bone mass. Obesity changes the bone microenvironment, inhibits the differentiation of bone marrow mesenchymal stem cells (BMSCs) into osteoblasts, promotes adipogenic differentiation, and increases bone fat. The high inflammatory state caused by obesity and the secretion of adipokines also have a negative effect on bone (Forte et al., 2023; Piñar-Gutierrez et al., 2022; Shapses and Sukumar, 2012).

Regular physical exercises are conducive to physical health, and weight-bearing exercises are crucial to bone metabolism and homeostasis. Mechanical stress generated by exercise and the secretion of related factors can promote bone formation, reduce bone resorption, improve bone mass, and thus reduce the risk of falls (Qiu et al., 2023; Styner et al., 2014). Exercise can alleviate the acquisition of bone marrow adipose tissue, and promote the formation of bone mass in obese mice (Chubak et al., 2006). Although proper exercise can promote bone mass, the molecular mechanism of aerobic exercise-induced bone remodeling in obese individuals has not been elucidated. In the present study, we used high-fat diet (HFD) to build a mouse obese model and identified their bone phenotype. The obese male mice underwent a 10-week treadmill exercise to explore the effects of aerobic exercise on the bones and identify the related mechanisms. Results will provide exercise reference for children and adolescents with bone mass loss due to obesity.

## Materials and methods

### Ethics statement

This study was carried out in strict accordance with the recommendations from the Ethical Committee of School of Physical Education and Sports Science, South China Normal University on the Care and Use of Animal Subjects in Research (Approval Number: SCNU-SPT-2020-003). Five mice were housed in each cage and were provided water *ad libitum*. All mice were maintained in an environment with a 12-h light–dark cycle (7:00-19:00) at 22 °C ± 2 °C.

### Treatment of animals

Sixty 5-week-old C57BL/6 male mice were randomly divided into normal diet group (CTR group, n = 30) and high fat diet group (HFD group, n = 30) for 12 weeks. The CTR group was given common feed, while the HFD group was administered with specially-made high-fat feed (D12492, Sailing Organism). The weight of mice in each group was recorded every week. The difference in the area under curve (AUC) between glucose tolerance test (GTT) and insulin tolerance test (ITT) in the HFD group was used to judge the success of modeling. We selected the heaviest 21 mice as obese male mice and randomly divided them into the obese group (HFD group, *n* = 9) and the obese exercise group (HFD + EX group, *n* = 12) according to their weight. Twenty-two mice given with ordinary diet were randomly divided into the control group (CTR group, *n* = 10) and the exercise control group (CTR + EX group, *n* = 12) according to their weight. Theses mice were then given exercise intervention for 10 weeks and provided with the same feed. At 5 weeks of age, mice were subjected to a 12 weeks high-fat diet to induce an obesity model; at 17 weeks of age, the mice began a 10-week treadmill intervention; finally, the mice were euthanized and samples were collected at 27 weeks of age, because after the end of the exercise intervention at 26 weeks of age, indicator tests will be conducted on the mice, such as Glucose Tolerance Test (GTT) and Insulin Tolerance Test (ITT).

### Treadmill running protocol

The treadmill running protocol is presented in Table 1. The first week was set for adaptive training. The running speed was gradually increased from 6 m/min to 10 m/min, the running slope was 12, and the training duration was 50min each time for 5 days a week. After the adaptive training, the running speed was increased by 1 m/min every week until 15 m/min, at which it was kept the same with a gradient of 12, training for 50 min every time and training for 5 days every week. To control for circadian effects, all running training sessions were conducted daily at the same time, starting at zeitgeber time 14 (ZT14). Given that ZT0 is defined as the time of lights-on (07:00) in our facility, ZT14 corresponds to 14 h after lights-on, which is 21:00.

**TABLE 1:** Treadmill running protocol.

Time weeks	Friday	Saturday	Sunday	Monday	Tuesday	Wednesday/Thursday
1	6 m/min, 50min, 12°	7 m/min, 50min, 12°	8 m/min, 50min,12°	9 m/min, 50min, 12°	10 m/min, 50min, 12°	Rest
2	11 m/min, 50min, 12°					
3	12 m/min, 50min, 12°					
4	13 m/min, 50min, 12°					
5	14 m/min, 50min, 12°					
6–10	15 m/min, 50min, 12°					

### Extraction, culture and stretch stimulation of primary BMSCs

Obese male mice were induced with HFD. BMSCs were extracted from both legs of mice in the CTR and HFD groups by conventional methods. After being treated with red cell lysates, BMSCs were planted in a 10 cm Petri dish and incubated for 7 days until BMSCs adhered to the wall. The α-MEM medium (containing 10% FBS+1% PS) was changed every other day, and the cells were cultured in an incubator at 37 °C and with 5% CO_2_. When the cells were full, they were digested with 0.25% trypsin, subcultured to the first generation, and inoculated into a six-well plate pulled by BioFlex at a rate of 1 × 10^5^/mL.

After the cells grew to 80%, they were subjected to tension stimulation using the Flexcell FX-5000 cell tension instrument. After intervention, the BMSCs of the CTR group and HFD groups were divided into four groups: control group (CTR), stretch control group (CTR + Str), obese control group (HFD), and obese stretch group (HFD + Str), with 6% tensile intensity, 0.5 Hz frequency, and the 4 h/day intervention time for 3 days. After the tension, cells were collected.

### Glucose tolerance test (GTT) and insulin tolerance test (ITT)

GTT: Before the experiment, the mice were fasted for 16 h, and 50% glucose solution was injected with 4 g/kg glucose intraperitoneally according to their weight. The blood glucose values of the tail vein were recorded at 0, 15, 30, 60, 90 and 120 min after injection. ITT: Before the experiment, the mice were fasted. According to the weight of the mice, 1 μL/kg insulin was injected intraperitoneally. The blood glucose values of the tail vein were recorded at 0, 15, 30, 60, 90 and 120min after injection.

### Micro-computed tomography measurements

The microarchitecture of the distal femoral metaphysis was analyzed using a VIVA CT80X micro-imaging system (SCANCO Medical AG, Switzerland). Each sample was scanned strictly in accordance with the following scanning parameters: 55 kVp, 145 μA, 8 W X-rays, and 9 μm. After the scan was completed, the reconstructed results were converted to TIFF format and then analyzed using the analysis software including Data Viewer, CTAn, and CTvox. To ensure complete comparability of the analyzed volumes between groups, we selected a standardized volume of interest (VOI) for each femur based on clear anatomical landmarks.For cancellous bone analysis, the starting position of the VOI was set exactly 0.5 mm proximal to the growth plate to exclude the primary spongiosa, and extended 1.0 mm proximally. This VOI was manually outlined to include only the secondary spongiosa, with the cortex excluded. For cortical bone analysis, a 1.0 mm long cortical bone VOI was selected at the mid-diaphysis of the femur. This anatomical landmark-based method ensures that the identical anatomical region is analyzed for each sample, eliminating any potential bias in VOI selection. The microarchitecture parameters of the cortical bone and the trabecular bone were measured using built-in software and include the following: relative volume fraction of trabecular bone (BV/TV), number of trabecular bones (Tb.N), trabecular thickness (Tb.Th), degree of trabecular bone separation (Tb.Sp), and cortical thickness (Ct.Th).

### Calcein injection and bone histomorphometry

Mouse bones were labeled with subcutaneously injected calcein (5 μL/g) on days 1 and 8 before they were killed on day 9. The femurs injected with calcein in each group were fixed with 4% PFA. After gradient dehydration with alcohol and xylene, the samples were embedded in methyl methacrylate, solidified, and ground into squares. A Leica hard tissue slicer was used to collect samples at 4 μm. The slices were dried and stained with Masson trichromatic dye. The distance between the cancellous bone area and the lower edge of the growth plate of the distal femur was measured as 1–4 mm by using the digital morphometry system (high-resolution color subsystem of Osteomeasure). The measurement parameters were cancellous bone mineralization rate (MAR), bone formation rate/bone surface (BFR/BS), bone formation rate/bone volume (BFR/BV), bone formation rate/total tissue volume (BFR/TV), BV/TV, Tb.N, Tb.Th, and Tb.Sp as well as number and surface percentage of osteoblasts (N.Ob/B.Pm, N.Ob/T.Ar, Ob/BS) and osteoclasts (N.Oc/B.Pm, N.Oc/T.Ar, Oc/BS).

### H&E and TRACP staining

The femur fixed with 4% PFA was decalcified, dehydrated by xylene and gradient alcohol, and embedded in paraffin. A Leica tissue microtome was used to collect samples at 4 μm. For HE staining, the sample was dewaxed with xylene, hydrated with gradient alcohol, washed with distilled water, and dyed with hematoxylin for 5 min. The stained sample was washed with running water and subjected to hydrochloric acid alcohol differentiation for several seconds. The sample was washed again with running water, stained with eosin for 5 min, dehydrated with gradient alcohol, and sealed with neutral gum after passing xylene. Samples were selected for TRACP dyeing, dewaxed with xylene, hydrated with gradient alcohol, washed with distilled water, stained with TRACP kit, and sealed with neutral gum after passing xylene. The slices were observed under a microscope.

### RNA sequencing

Trizol (AG RNAex Pro RNA) was used to isolate the total RNA of the tibia of mice in each group. RNA-Seq library construction and RNA high-throughput sequencing were carried out by Beijing Genome Research Institute (BGI, China) on BGISEQ-500 high-throughput sequencing instrument. Gene ontology (GO), Kyoto Encyclopedia of Genes and Genomes (KEGG), Gene Set Enrichment Analysis (GSEA), Weighted Correlation Network Analysis (WGCNA), and Time Process Analysis were analyzed according to the previously described methods.

### Real-time quantitative PCR

Total RNA was isolated from the BMSCs or the left tibias of mice and reverse-transcribed using 1 µg of total RNA with oligo-dT primers at 42 °C for 1 h. All PCRs were performed using 2 µg of respective cDNA with SYBR Green qPCR Master Mix (Roche, Switzerland). *Alkaline phosphatase (ALP), Runt-related transcription factor 2 (Runx2)*, *activating transcription factor 4 (ATF4)*, *osteopontin (OPN)*, *cathepsin K (CTSK)*, *matrix metalloproteinase-9 (MMP9)*, *nuclear factor-activated T cell 1 (NFATc1)*, *cluster of differentiation 24 (CD24)*, *fatty acid transport protein 1 (Fatp1)*, *CCAAT/enhancer binding protein β (CEBPβ)*, and *peroxisome proliferator-activated receptor γ (PPARγ)* mRNA were measured by quantitative testing. Quantification of mRNA was performed using Step One Plus™ Sequencing Detection System (Life Technologies, United States). The following cycling parameters were used: 40 cycles of denaturation at 95 °C for 15 s and annealing at 60 °C for 60 s. The mouse primers used in this study are presented in Table 2.

**TABLE 2:** RT-PCR primer sequences.

mRNA	Primer	Sequences (5′– 3′)
ALP	Forward	CGGGACTGGTACTCGGATAA
	Reverse	ATTCCACGTCGGTTCTGTTC
Runx2	Forward	TTTAGGGCGCATTCCTCATC
	Reverse	TGTCCTTGTGGATTAAAAGGACTTG
ATF4	Forward	AAGGAGGAAGACACTCCCTCT
	Reverse	CAGGTGGGTCATAAGGTTTGG
OPN	Forward	AGCCACAAGTTTCACAGCCACAAGG
	Reverse	TGAGAAATGAGCAGTTAGTATTCCTGC
CTSK	Forward	CTCGGCGTTTAATTTGGGAGA
	Reverse	TCGAGAGGGAGGTATTCTGAGT
MMP9	Forward	CTGGACAGCCAGACACTAAAG
	Reverse	CTCGCGGCAAGTCTTCAGAG
NFATc1	Forward	CAACGCCCTGACCACCGATAG
	Reverse	GGCTGCCTTCCGTCTCATAGT
CD24	Forward	CAACGGGCTGCTATGGATTG
	Reverse	GCAGAGGTAGCCATCGACAG
Fapt1	Forward	GGCTGGGGCTAAGAATCCG
	Reverse	GCATACTCGTTCACTGGACAC
CEBPβ	Forward	TGTTCCTGCGGGGTTGTTGAT
	Reverse	CGAAACGGAAAAGGTTCTCAA
PPARγ	Forward	ACTGCCTATGAGCTCTTCAC
	Reverse	CAATCGGATGGTTCTTCGGA
HPRT	Forward	GTTAAGCAGTACAGCCCC AAA
	Reverse	AGGGCATATCCAACAACAAACTT

### Western blot assay

Total proteins were extracted from other tibias with RIPA Lysis Buffer (Biyuntian, China) containing a protease inhibitor cocktail (Roche, Switzerland). The proteins were separated by 10% SDS-PAGE and transferred onto a 0.45 µm PVDF membrane (Millipore, United States). The blotting membranes were blocked with 5% skim milk powder in 1×TBS-Tween (TBST) for 1 h. After washing with 1×TBST three times for 10 min each, the blotting membranes incubated with rabbit polyclonal Runx2 (1:1,000; CST, #12556), rabbit polyclonal CTSK (1:1,000; CST, #57056), rabbit polyclonal CCN4 (1:1,000; Abcam, ab260036), and rabbit polyclonal GAPDH antibody (1:1,000; Cell Signaling Technology, #2118S).

### Immunofluorescence

The femur fixed with 4% paraformaldehyde was embedded with OCT, frozen, sectioned with a thickness of 10 μm, and then stained with immunofluorescence. The sample was washed with PBS three times, blocked with 1% bovine serum albumin and 0.3% Triton X-100 for 30 min, and incubated with the primary antibody (OCN, 1:200; Proteintech, GB11233-100) at 4 °C overnight. The sample was incubated with the second antibody (AF488-labeled Goat Anti-Rabbit lgG (H + L), 1:2000; Beyotime, A0423) for 2 h the next day. Anti-fluorescence quenching sealing solution containing DAPI were dripped, and images were obtained with Leica confocal imaging system.

### Analysis of serum biochemical markers

Serum insulin (INS), triglycerides (TG), total cholesterol (CHOL), and free fatty acids (NEFA) were detected. Serum samples were obtained from six mice from each group, and the corresponding indicators were detected by radioimmunoassay.

The levels of serum ALP, procollagen type I N-terminal propeptide (PINP), serum calcium (Ca), and collagen cross-linked terminal peptide (CTX-1) of collagen type I were determined by enzyme-linked immunosorbent assay kit (China Yunclone Company).

### Statistical analysis

All data were processed by SPSS26.0 software, and the results were expressed by mean ± SEM. Two-way ANOVA was used to analyze the presence of any difference between the groups. *P < 0.05* was considered statistically significant.

## Results

### Aerobic treadmill exercise reduced the body weight of obese male mice and alleviated abnormal blood glucose in obese male mice

**FIGURE 1:**
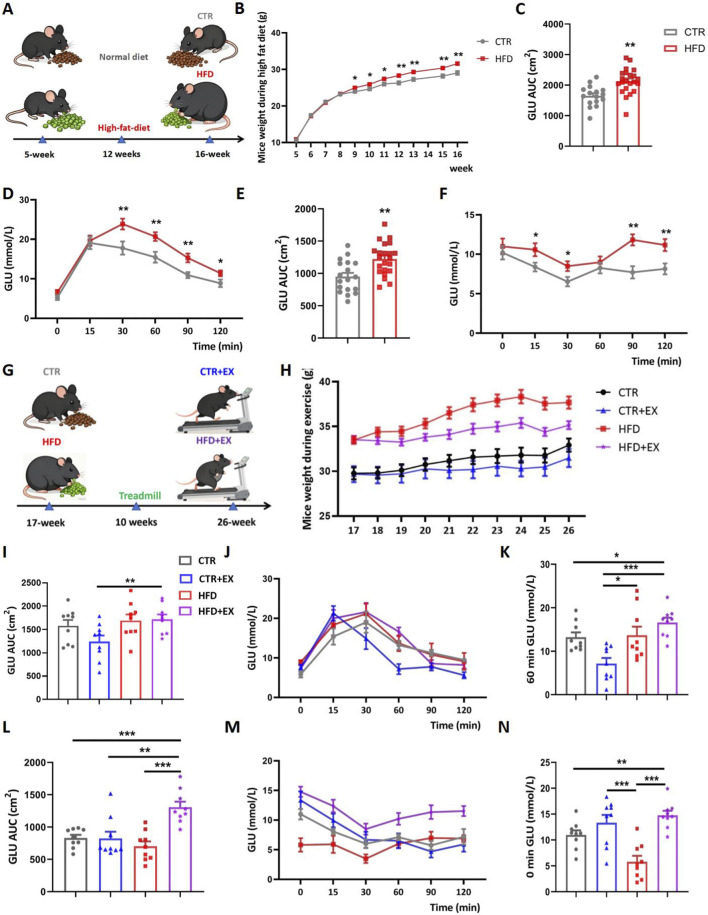
Aerobic treadmill exercise reduces the body weight of obese male mice and alleviated abnormal blood glucose in obese male mice. **(A)** HFD was given to establish obesity model; **(B)** weight change of mice during obesity modeling period (CTR, n = 19; HFD, n = 22); **(C)** GTT AUC of mice after 3 months of high-fat diet feeding (CTR, n = 16; HFD, n = 22); **(D)** GTT of mouse blood glucose values in different time periods (CTR, n = 16; HFD, n = 22); **(E)** ITT AUC of mice after 3 months of high-fat diet feeding (CTR, n = 18; HFD, n = 22); **(F)** ITT of mice blood glucose values in different time periods (CTR, n = 16; HFD, n = 22); **(G)** Aerobic treadmill exercise program for mice; **(H)** Mouse weight changes during exercise (CTR, n = 10; HFD, n = 9; CTR + EX, n = 12; HFD + EX, n = 12); **(I)** GTT AUC of obese male mice after 10 weeks of exercise (n = 9); **(J)** Blood glucose values of mice in different periods of GTT after 10 weeks of exercise (n = 9); **(K)** Changes in blood glucose in mice in each group at 60 min after glucose injection (n = 9); **(L)** ITT AUC of obese male mice after 10 weeks of exercise (n = 9); **(M)** Blood glucose values of mice in different periods of ITT after 10 weeks of exercise (n = 9); **(N)** Changes of blood glucose in each group immediately after insulin injection (n = 9). *P < 0.05, **P < 0.01, ***P < 0.001. Each data point represents an independent biological sample. Bars represent mean ± SEM.

Figure 1 summarizes the basic physiological parameters of mice after intervention. The experimental intervention timeline of the mice (Figure 1A). We first evaluated the effect of HFD on body weight, as shown in (Figure 1B), the body weight growth curves of mice in each group indicate that a difference in body weight between the CTR group and HFD group emerged at the fifth week of high-fat diet feeding. In addition, we conducted a comprehensive assessment of systemic metabolic phenotypes in each group and found that high-fat diet impaired glucose tolerance in the HFD group, which was manifested by an increased area under the curve (AUC) value in the GTT (Figures 1C,D). This was accompanied by significant insulin resistance, as reflected by a marked increase in the AUC value in the ITT (Figures 1E,F). We randomly divided the CTR group into the CTR group and the CTR + EX group, and divided the HFD group into the HFD group and the HFD + EX group. Mice in the exercise group underwent aerobic treadmill training for 10 weeks, during which the feed of each group remained unchanged (Figure 1G). From the 21st week, the weight of mice in the HFD + EX group was significantly lower than that in the HFD group (Supplementary Figure S1). Aerobic treadmill exercise for 10 weeks can significantly reduce the weight of obese male mice. We also conducted glucose tolerance and insulin tolerance tests. Compared with CTR mice, CTR + EX mice had better glucose and insulin tolerance. Obesity can cause impaired glucose and insulin tolerance in mice, and 10 weeks of aerobic exercise intervention cannot restore glucose tolerance and insulin tolerance (Figures 1I–N).

### Aerobic treadmill exercise improves lipid metabolism in obese male mice

**FIGURE 2:**
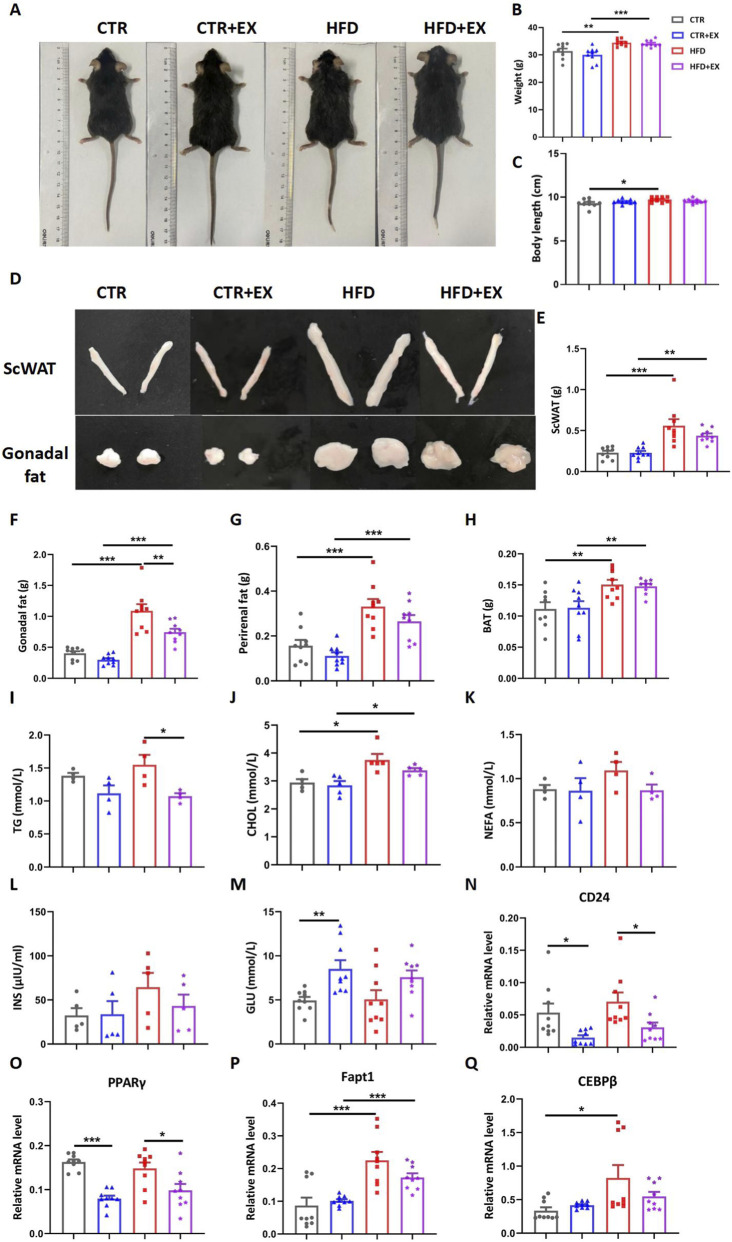
Aerobic treadmill exercise improves lipid metabolism in obese male mice. **(A)** Image of mice after 3 months of high-fat dietary feeding; **(B)** Body weight of mice in each group (n = 9); **(C)** Length of mice in each group (n = 9); **(D)** Image of ScWAT and Gonadal fat in mice after 3 months of high-fat dietary feeding; **(E–H)** Weight of ScWAT, Gonadal fat, Perirenal fat, and BAT in each group (n = 9); **(I–L)** Serum TG (n = 5), total CHOL (n = 4), NEFA (n = 4), and INS (n = 5) concentrations of mice in each group; **(M)** Resting blood glucose content of mice in each group (n = 9); **(N–Q)** Relative mRNA levels of CD24, PPARγ, Fapt1, and CEBPβ in mice of each group (n = 9). *P < 0.05, **P < 0.01, ***P < 0.001. Each data point represents an independent biological sample. Bars represent mean ± SEM.

Before sacrifice, all mice were anesthetized, and photos of their overall morphology were taken (Figure 2A). Subsequently, the body weight of mice in each group was measured. Compared with the CTR group, the body weight of mice in the high-fat HFD group significantly increased; despite treadmill exercise intervention, the body weight of mice in the HFD group remained significantly higher than that of CTR + EX group (Figure 2B). Furthermore, compared with the CTR group, the body length of mice in the HFD group also showed a trend of being slightly longer than that of the control group (Figure 2C). The Subcutaneous Adipose Tissue (ScWAT), the gonadal fat, the perirenal fat, and the brown adipose tissue (BAT) of mice were isolated after dissection, photographed, and weighed (Figures 2D–H). The weight, body length, and four kinds of adipose tissues of mice in the HFD group were significantly higher than those in the CTR group. After 10 weeks of treadmill exercise, the gonadal fat weight of the HFD + EX group was significantly lower than that of the HFD group, so 10 weeks of aerobic treadmill exercise could reduce the gonadal fat weight of obese male mice.

Radioimmunoassay was used to detect lipid metabolism in the serum of mice. The serum TG level of mice in the HFD group was reduced by treadmill exercise for 10 weeks (Figure 2I). Obesity increased serum CHOL level in mice (Figure 2J). Obesity and exercise had no significant difference in the serum NEFA and INS levels of mice, but the trend is consistent (Figures 2K,L). However, the blood glucose of mice in the CTR + EX group was significantly higher than that in the CTR group (Figure 2M).

After isolating mouse bones, RT-PCR was used to detect the relative mRNA levels of fat gene markers in mouse bones. The mRNA of *Fapt1* and *CEBPβ* in the HFD group was significantly higher than that in the CTR group. The 10-week aerobic treadmill exercise significantly reduced the mRNA of *CD24* and *PPARγ* in the CTR and HFD groups (Figures 2N–Q). Therefore, the weight gain and fat content of obese male mice models induced by HFD can be effectively alleviated by aerobic exercises.

### Aerobic treadmill exercise alleviates the decrease of bone mass in obese male mice

**FIGURE 3:**
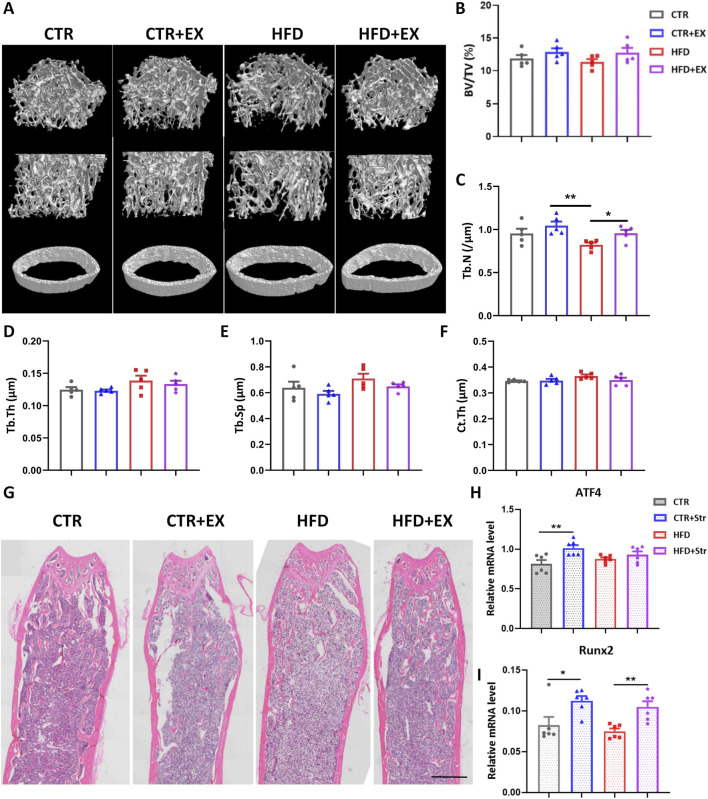
Aerobic treadmill exercise alleviates the decrease of bone mass in obese male mice. **(A)** Micro-CT 3D reconstruction of cancellous bone and cortical bone of distal femur; **(B–F)** BV/TV, Tb.N, Tb.Th, Tb.Sp and Ct.Th of mice in each group (n = 5); **(G)** HE staining diagram of mice in each group; **(H,I)** Levels of *ATF4* and *Runx2* mRNA in obese male mice after BMSCs mechanical traction (n = 6). ^*^ *P < 0.05,* ^**^ *P < 0.01.* Each data point represents an independent biological sample. Bars represent mean ± SEM.

To determine the effect of aerobic exercise on the bones of obese male mice, we performed micro-CT tomography and 3D reconstruction analysis of the femurs in each group of mice (Figure 3A). 10 weeks of aerobic exercise improved bone mass loss (Figure 3C), and differences in BV/TV, Tb.Th, Tb.Sp, and Ct.Th were not significant (Figures 3B,D–F). HE staining showed that compared with the CTR group, the cancellous bone space in the HFD group was wider, and the trabecular bone was less. However, the trabecular bone of mice in HFD + EX group increased significantly (Figure 3G).

In addition, we isolated the femurs and tibia of 12-week obese male mice and their control mice, extracted BMSCs, seeded in a six-well traction plate, and simulated mechanical stimulation *in vitro* using a cellular mechanical traction device. After stretching BMSCs, RNA was extracted from cells in each group. RT-PCR results showed that the expression of osteogenic genes *ATF4* and *Runx2* mRNA in BMSCs increased in the CTR + Str group. The expression of *Runx2* mRNA in the BMSCs in HFD + Str group was significantly higher than that in the HFD group (Figures 3H,I).

### Effect of aerobic treadmill exercise on bone formation in obese male mice

**FIGURE 4:**
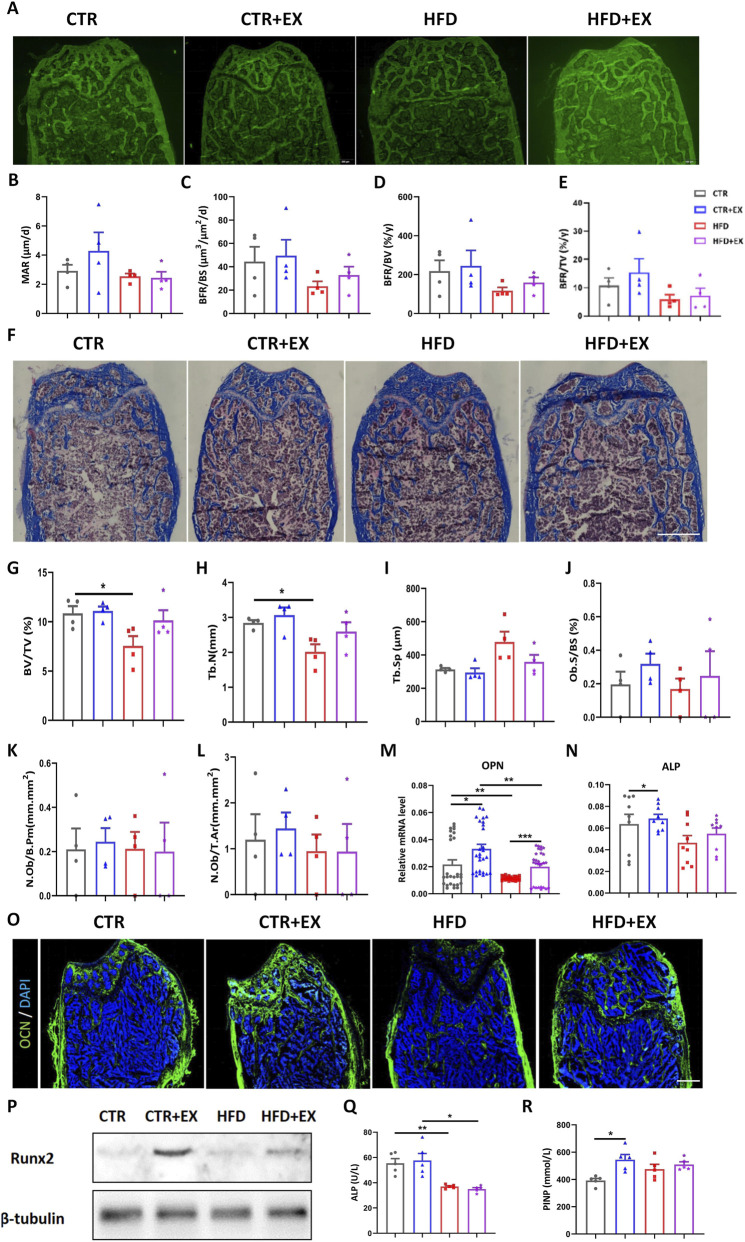
Aerobic treadmill exercise promotes bone formation and alleviates bone loss in obese male mice. **(A)** Image of calcein-labeled bones injected subcutaneously in mice 1 day and 8 days before sacrifice; **(B–E)** MAR, BFR/BS, BFR/BV, BFR/TV of mice in each group (n = 4). **(F)** Image of Masson staining of femoral hard tissue sections of mice in each group; **(G–L)** BV/TV, Tb.N, Tb.Sp, Ob.S/BS, N.Ob/B.Pm, N.Ob/T.Ar in each group of mice (n = 4). **(M,N)** The mRNA levels of tibial *OPN* and *ALP* in each group of mice (n = 9); **(O)** Immunofluorescence staining of femoral OCN protein in each group: green (OCN) and blue (DAPI); **(P)** Expression of runx2 protein in tibia of mice in each group; **(Q,R)** Serum levels of ALP and PINP in mice in each group (n = 5). ^*^ *P < 0.05,* ^**^ *P < 0.01.* Each data point represents an independent biological sample. Bars represent mean ± SEM.

To further investigate the effect of aerobic treadmill exercise on bone formation in obese male mice, we labeled the bones of mice with subcutaneous calcein (5 μL/g) 1 and 8 days before sacrifice. Bone histomorphometrics were used after resin embedding to make hard bone sections (Figure 4A). Compared with the three other groups, the femoral MAR and BFR of the HFD group were the lowest among the other groups, although the differences among the groups was not significant. Although no significant differences were found in femoral MAR and BFR in the CTR + EX group and other groups, and the trend was increasing (Figures 4B–E). In the state of obesity, aerobic exercise can promote the rate of bone formation to a certain extent. After Masson three-color staining of bony sections (Figure 4F), the femur BV/TV and Tb.N of mice in the HFD group was lower than those in the CTR group (Figures 4G,H). No significant difference in bone formation rate was found among the other groups. Obesity inhibited femoral bone formation rate, and treadmill exercise could increase the trend of bone formation in obese male mice to a certain extent (Figures 4I–L).

The mRNA expression of *OPN* in the tibia of mice in the HFD group was significantly lower than that in the CTR group, and the mRNA expression of *ALP* and *OPN* in the tibia of mice in the CTR + EX group was higher than that in the CTR group. The mRNA expression of *OPN* could be increased by aerobic treadmill exercise for 10 weeks (Figures 4M,N). Immunofluorescence and Western blot results showed that 10 weeks of aerobic treadmill exercise increased the protein content of OCN and Runx2 (Figures 4O,P).

Furthermore, obesity induced by HFD led to a significant decrease in ALP content in mouse serum (Figure 4Q), while 10 weeks of aerobic treadmill exercise increased PINP content (Figure 4R).

### Effects of aerobic treadmill exercise on bone resorption in obese male mice

**FIGURE 5:**
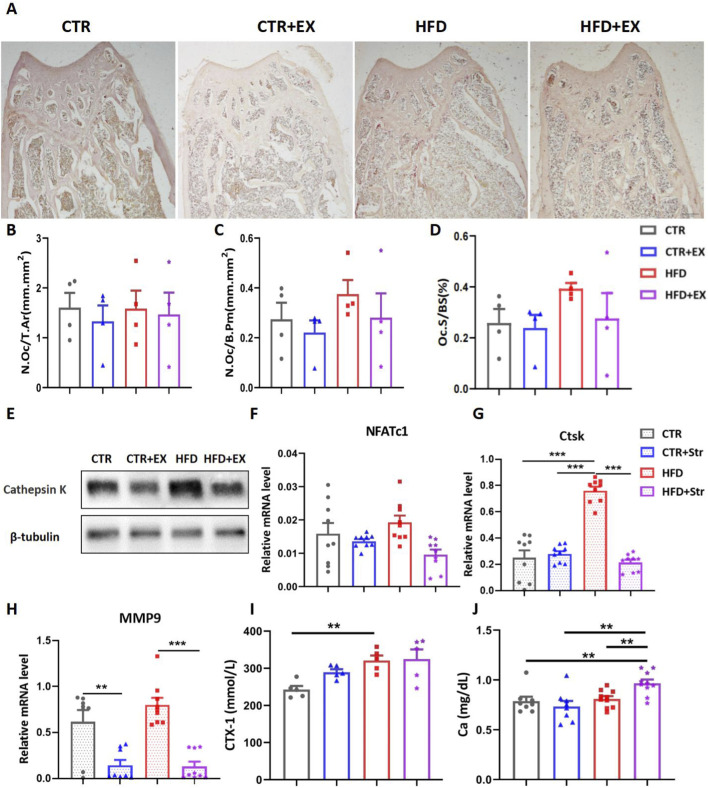
Aerobic treadmill exercise inhibits bone resorption in obese male mice. **(A)** Image of TRACP staining in mice in each group; **(B–D)** bone morphometry was used to detect the number of femoral N.Oc/T.AR and the area of N.Oc/B.Pm, Oc.S/BS in each group (n = 4); **(E)** Ctsk protein expression in tibias of mice from each group; **(F)** *NFATc1* mRNA levels in tibias of mice from each group (n = 9); **(G,H)** *Ctsk* and *MMP9* mRNA levels after mechanical stretching of BMSCs from obese male mice (n = 9); **(I)** Urinary CTX-1 levels in mice from each group (n = 5); **(J)** Serum Ca levels in mice from each group (CTR, n = 8; HFD, n = 8; CTR + EX, n = 10; HFD + EX, n = 10). ^**^ *P < 0.01,* ^***^ *P < 0.001.* Each data point represents an independent biological sample. Bars represent mean ± SEM.

To further investigate the effects of aerobic treadmill exercise on bone resorption in obese male mice, we performed TRACP staining. Mice in the HFD group showed increased bone resorption in trabecular bone, while exercise mitigated this phenomenon (Figure 5A). Bone histomorphometry statistics showed that obesity induced by HFD tended to increase bone resorption indices in mice. Aerobic treadmill exercise mitigated this trend. However, no significant differences were observed among the groups (Figures 5B–D). Western blot results showed that the Ctsk protein was significantly elevated in the HFD group, while 10 weeks of aerobic treadmill exercise inhibited the increase in Ctsk (Figure 5E). Although the expression of *NFATc1* mRNA in the tibias was not significantly different among the groups, exercise showed a trend of alleviating bone resorption in obese male mice (Figure 5F). After stretching the BMSCs, RNA was extracted from each group. The RT-PCR results showed that stretching effectively inhibited the upregulation of mouse osteoclast genes Ctsk and MMP9 induced by obesity (Figures 5G,H). In addition, we detected the expression levels of urinary bone resorption marker CTX-1 and serum Ca in mice. The urinary CTX-1 content in the HFD group mice was higher than that in the CTR group (Figure 5I). The serum Ca content in the HFD+EX group mice was significantly higher than that in the CTR, CTR+EX and HFD groups (Figure 5J).

### Obesity induced by HFD inhibits WNT signal transduction in mouse bones

**FIGURE 6:**
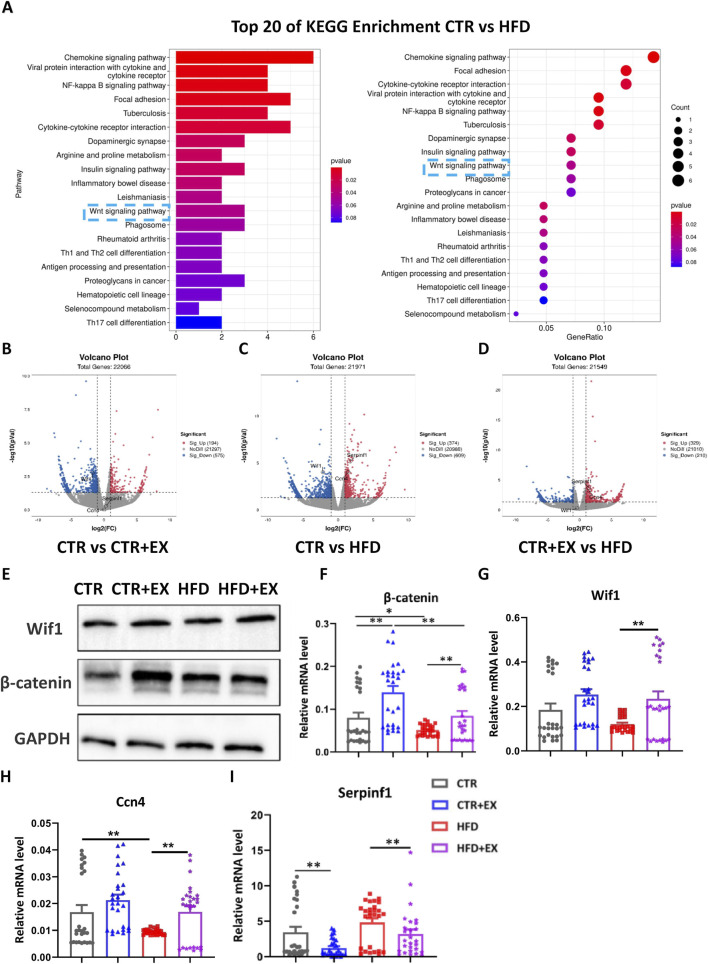
Obesity induced by high-fat diet inhibits WNT signal transduction in male mice bones. **(A)** Top 20 significantly enriched KEGG Biological Process of HFD male mice models, P < 0.05; **(B–D)** Respective volcano plots of sifted out DEGs for datasets of CTR and EX, CTR and HFD, EX and HFD mice tibias. Blue and red plots respectively represent down- and upregulated genes. Gray plots represent the remaining genes with no significant difference; **(E)** β-catenin and Wif1 protein expression in the tibias of mice from each group; **(F–I)** mRNA expression levels of *β-catenin*, *Wif1*, *Ccn4*, and *Serpinf1* in the tibias of mice from each group. ^*^ *P < 0.05,* ^**^ *P < 0.01,* ^***^ *P < 0.001.* Each data point represents an independent biological sample. Bars represent mean ± SEM.

RNA-Seq detection and KEGG pathway analysis of mouse tibias showed that obesity induced by HFD inhibited the WNT signaling pathway (Figure 6A). Among 22,066 differentially expressed genes, the expression of Wif1 was decreased in the CTR + EX group compared with that in the CTR group (Figure 6B). Among 21,971 differentially expressed genes, the expression of Serpinf1 and Ccn4 was upregulated in the HFD group compared with that in the CTR group, while the expression of Wif1 was downregulated compared with that in the CTR group (Figure 6C). Among 21,549 differentially expressed genes, the expression of Serpinf1 and Ccn4 was upregulated in the HFD group compared with tha in the CTR + EX group (Figure 6D). Total protein was extracted from the tibias of mice in each group. The Western blot results showed that the expression levels of Wif1 and β-catenin proteins increased in the CTR + EX group, while that of the β-catenin protein was significantly inhibited in the HFD group compared with those in the CTR group (Figure 6E). Total RNA was subsequently extracted from the tibias of mice form each group. The RT-PCR results showed that HFD decreased *Ccn4* mRNA expression. Ten weeks of aerobic treadmill exercise effectively alleviated the decrease in *β-catenin*, *Wif1*, and *Ccn4* mRNA expression caused by HFD, while *Serpinf1* mRNA showed the opposite trend (Figures 6F–I). These results suggest that obesity induced by HFD may lead to bone loss by inhibiting the WNT signaling pathway.

## Discussion

The main findings of this study can be summarized as follows. Obesity induced by HFD inhibits bone formation, promotes bone resorption, and leads to decreased bone mass in obese male mice. The effects of HFD-induced obesity on the bones are mainly associated with the inhibition of the WNT signaling pathway and the dysregulation of adipokine production. Aerobic exercise can, to some extent, alleviate the inhibition of WNT pathway transmission caused by obesity, promote bone formation, and inhibit bone resorption.

Male mice were selected to avoid the significant interference of the estrogen cycle on bone metabolism. Bone metabolism, especially bone formation, is strongly regulated by estrogen, and the estrogen level in female mice fluctuates regularly with the estrous cycle (usually 4–5 days) (Almeida et al., 2017; Xiong and O'Brien, 2012). The periodic fluctuations of such endogenous hormones are themselves a powerful and difficult to control variable, which can significantly affect the baseline status and response of osteoblast activity, osteoclast formation, and key signaling pathways such as Wnt/β-catenin (Melville et al., 2014). Therefore, in the initial exploratory study of mechanisms, prioritizing the use of male animals with a relatively stable physiological hormone environment is an effective strategy to control variables and ensure that we can clearly observe the core causal chain between exercise and bone (Beery and Zucker, 2011).

Obesity not only increases the risk of chronic metabolic diseases, such as diabetes and hypertension, but is also closely related to skeletal health (Klein-Nulend et al., 1995). Mechanical load is one of the factors contributing to increased bone mass. Therefore, the increased body weight caused by obesity was thought to apply additional force to the body, thereby increasing bone mass to some extent and reducing the occurrence of OP (Gower and Casazza, 2013; Guo et al., 2021). However, in recent years, an increasing number of studies have confirmed that obesity leads to fat accumulation and trabecular bone loss (Kleinert et al., 2018). The mechanisms involved include changes in bone cell metabolism, increased oxidative stress and inflammation, and alterations in bone-regulating hormones. Nevertheless, the mechanisms by which exercise improves obesity-induced bone loss require further investigation. Therefore, we designed this study to examine changes in bone mass in obese male mice due to exercise and explore the related bone turnover mechanism.

Feeding mice with a diet containing 60% fat resulted in a significant increase in body weight, elevated serum levels of TG, CHOL, and INS, and decreased glucose tolerance and insulin resistance (Gonnelli et al., 2014; Thyfault and Rector, 2020). Exercise can increase the body’s energy consumption, thereby reducing body weight and lowering fasting insulin levels (Nielson et al., 2011). After feeding 5-week-old C57BL/6 mice with HFD containing 60% fat for 12 weeks, the body weight consistently remained higher than that of control mice, and the obese male mice exhibited decreased glucose tolerance and insulin resistance. After 10 weeks of aerobic treadmill exercise, the body weight and fat mass of the mice significantly decreased. However, in the results of Figure 2B, the inter-group difference failed to reach the significant level observed in Figure 1H. We noticed that in the last week of the experiment, the mice underwent short-term fasting and injections for GTT and ITT. These necessary experimental procedures inevitably caused acute stress to the mice and might have temporarily affected their feeding behavior and energy metabolism. In addition, Supplementary Figure S1 of ours includes the weekly body weight measured during the exercise period for reference. The GTT AUC values of the four groups of mice showed that glucose clearance rates increased after aerobic exercise. Additionally, exercise improved serum CHOL and TG levels in obese male mice. The RT-PCR detection revealed that exercise reduced the expression of obesity-related fat genes in obese male mice. Hence, aerobic treadmill exercise, to some extent, alleviated abnormal lipid metabolism in obese male mice induced by HFD.

To control for the potential impact of circadian rhythms on exercise benefits, all exercise interventions in this study were strictly standardized to be conducted within the same time window (ZT 14). Evidence indicates that the physiological responses of organisms to exercise, including changes in energy metabolism and skeletal adaptability, are regulated by the intrinsic circadian clock (Sc et al., 2015; Wolff and Esser, 2012). Recent studies have clearly indicated that exercising at different stages of the animal’s active phase (ZT12-24) exerts distinctly different effects on bone and metabolism (Boudenot et al., 2021; Yu et al., 2024) For instance, exercise performed in the early stage of the active phase may primarily promote anabolism, whereas exercise conducted during the resting phase could disrupt normal rhythms and induce a stress response (Sato et al., 2019). Therefore, standardizing the exercise time is crucial for obtaining consistent and reproducible experimental results. In this study, treadmill training was conducted in male mice during the early active phase (ZT14). This choice aimed to mimic their natural activity peak and standardize the exercise intervention as a variable as much as possible, thereby ensuring that the observed effects of promoting bone formation and activating the Wnt pathway can be clearly attributed to exercise itself, rather than confounding effects caused by differences in exercise timing.

Under normal circumstances, the amount of old bone absorbed by osteoclasts is consistent with the amount of new bones generated by osteoblasts. This balance maintains the continuous renewal of bone tissue and its mechanical integrity throughout life, playing an important role in maintaining the homeostasis of trace elements, such as calcium and phosphorus, in the body. However, under certain physiological or pathological conditions, the activity of osteoblasts and osteoclasts may change accordingly. When the two processes of bone metabolism are no longer balanced, bone tissue will exhibit corresponding changes, such as physiological growth, development, and aging or have pathological conditions, such as OP, osteomalacia, bone metastases, and others. Obese males often exhibit significant decreases in BMD, BV/TV, Tb.N, and Tb.Th (Kang et al., 2019; Kang et al., 2017). However, after exercise intervention, the adverse effects of obesity on bones can be mitigated. Kang et al. (Kang et al., 2017) subjected obese rats to 8 weeks of swimming training, and the micro-CT results showed significant increases in BMD, BV/TV, and Tb.N in the femur and tibia of obese rats after swimming. The results consistently demonstrate that exercise can alleviate bone loss caused by obesity regardless of treadmill running, voluntary wheel running, swimming, or ladder climbing resistance exercise (Styner et al., 2014; Kang et al., 2019; Shi et al., 2022; Yan et al., 2021). Our micro-CT, Masson staining, and bone fluorescence results demonstrate that obesity decreases bone mass and inhibits the rate of femoral bone formation in mice. However, aerobic treadmill exercise can increase the trend of bone formation in obese male mice. Additionally, the HE staining results of the femur in each group of mice show similar findings, that is, obese male mice have widened trabecular spaces and reduced trabecular bone. After aerobic exercise, the trabecular bone in obese male mice significantly increases. Furthermore, the serum bone formation marker ALP and the expression of osteogenic genes OPN and ALP mRNA as well as the OCN protein in the bone are significantly reduced. ALP is a classic early-stage marker of osteogenic differentiation. The increase in its activity indicates the differentiation commitment of progenitor cells toward the osteoblast lineage, reflecting the initial stage of bone matrix maturation (Nakamura et al., 2020). In contrast, OPN is regarded as a middle-to-late-stage marker of the osteogenic process. By analyzing the expression of OPN, we aim to evaluate the differentiation progress and functional maturation status of osteoblasts (Chen et al., 2014). However, 10 weeks of aerobic treadmill exercise can promote the expression of osteogenesis-related genes and proteins. After extracting BMSCs, mechanical stimulation was simulated *in vitro*. The RT-PCR results were consistent with the above findings, that is, *in vitro* stretching of obese mouse BMSCs could promote the expression of osteogenesis-related genes. The above results indicate that after treadmill exercise, bone mass increases, suggesting changes in bone metabolism levels, although the difference was not significant. The reasons may involve issues, such as exercise selection, sample and parameter choices, etc. This also suggests that we should consider these factors in our future research to further explore the relationship between exercise and the prevention of bone metabolism diseases caused by obesity.

TRACP staining and Masson’s trichrome staining results show that obesity induced by HFD tends to increase bone resorption indicators in mice, while aerobic treadmill exercise mitigates this trend. After aerobic exercise intervention, the expression of NFATC1 mRNA and the level of the ctsk protein in the tibia of obese male mice decreased significantly. Previous experimental results also showed that HFD upregulates bone resorption in mice, while treadmill exercise training can alleviate the expression of bone resorption (Cao and Picklo, 2015). Ca and P are essential minerals in the human body. They form the basic components of bones as phosphates and confer bones their appropriate strength. Only 1% of Ca is present in the blood. When the balance of Ca and P in the blood is disrupted, the body automatically mobilizes Ca and P stored in the skeletal system to regulate the balance (Blaine et al., 2015). This experiment found that blood Ca levels significantly increased in HFD mice and HFD + EX group mice. The elevated levels of CTX-1 in urine typically indicate increased rates of bone matrix degradation and bone resorption, which is common in diseases such as osteoporosis. In elderly individuals, a significant negative correlation was found between urinary CTX-1 concentration and the degree of obesity; obese patients had relatively lower levels of CTX-1 in their urine (Djafari et al., 2021). In this experiment, obesity significantly increased in urinary CTX-1 levels in mice. After extracting BMSCs, mechanical stimulation was simulated *in vitro*. The RT-PCR results were consistent with the above findings, namely, that *in vitro* stretching of obese male mice BMSCs could inhibit the expression of osteoclast-related genes. Hence, exercise can improve the function of osteoclasts induced by HFD in obese male mice and reduce bone resorption.

The classical WNT/β-catenin signaling pathway is one of the most conserved regulatory factors in evolution and plays a crucial role in regulating tissue development and maintaining homeostasis (Liu et al., 2022). Meanwhile, the Wnt/β-catenin signaling pathway is also a core regulator of bone metabolism. Studies have reported that the Wnt/β-catenin signaling pathway regulates the differentiation of MSCs into osteoclasts by inhibiting the adipogenic transcription factor C/EBPα and PPARγ, thereby playing a crucial role in the regulation of bone homeostasis. Additionally, the interaction between β-catenin and TCF (T-cell factor) proteins can upregulate the expression of OPG (Osteoprotegerin) in osteoclasts, which serves to block the differentiation of osteoclasts (Rodda and McMahon, 2006). In this study, RNA-seq, Western blot, and RT-PCR consistently confirmed that obesity induced by a high-fat diet significantly inhibited the Wnt/β-catenin signaling pathway in the tibia of mice, which was manifested by the downregulation of β-catenin and Ccn4. However, 10 weeks of aerobic treadmill exercise effectively reversed this inhibitory trend, as evidenced by the increased expression of β-catenin, Wif1, and Ccn4. We propose that the activation of skeletal Wnt signaling by exercise may be achieved through the synergistic effect of two aspects.

Exercise may safeguard the normal function of the Wnt pathway by improving the obesity-related systemic pathological environment. Obesity is a state of chronic inflammation, characterized by elevated levels of circulating inflammatory factors such as TNF-α and IL-6 (Hotamislig and il, 2006). These inflammatory factors are potent inhibitors of the Wnt pathway, as they can upregulate the expression of Wnt antagonists Dkk1 and sclerostin (Poole et al., 2005). Wannenes et al. (Wannenes et al., 2014).cultured osteoblasts by using serum from obese patients and found that the WNT pathway was inhibited, with its downstream target genes downregulated. When using palmitic acid (PA) to simulate high-fat-induced *in vitro* obesity state, the expression levels of WNT3a and β-catenin were significantly decreased in the PA group compared with those in the control group (Guo et al., 2022). This indicates that obesity itself directly inhibits the Wnt pathway. Therefore, we hypothesize that aerobic exercise, as an effective anti-inflammatory intervention (Pedersen and Saltin, 2015), may reduce the induction of Dkk1/sclerostin by lowering the inflammatory levels in the whole body and local bones, thereby indirectly relieving the inhibition on the Wnt pathway and enabling the recovery of β-catenin signaling. Although this study did not directly detect Sost, the observed change in the expression of Wif1 (another antagonist) in the exercise group suggests that the regulation of antagonists may be a core mechanism. Future studies that directly detect changes in the levels of inflammatory factors and Sost protein in serum and bone tissue after exercise will strongly validate this hypothesis.

The mechanical load exerted by exercise on bones may be a direct pathway. Bones are highly mechanically sensitive tissues, In clinical settings, strength training can upregulate the expression of WNT-related genes in the human body (Cardaci et al., 2020), and mechanical stimulation can directly inhibit the expression of Sost in osteocytes (Robling et al., 2008; Tu et al., 2012)while promoting the secretion of Wnt ligands by osteoblasts (Lawson et al., 2022). We detected changes in Ccn4 (WISP1). As a downstream target gene and secretory protein of the Wnt pathway, Ccn4 itself can enhance the differentiation and proliferation of osteoblasts and inhibit adipogenesis through autocrine/paracrine mechanisms (Zuo et al., 2010). Therefore, the upregulation of Ccn4 by exercise is not only a response to the activation of the Wnt pathway, but also likely constitutes a positive feedback loop itself, which further amplifies the osteogenic effect of exercise.In summary, under the state of obesity induced by a high-fat diet, factors such as chronic inflammation lead to the inhibition of the Wnt pathway and a reduction in bone formation. On one hand, aerobic treadmill exercise reduces the inhibition of the pathway through its systemic anti-inflammatory effect. On the other hand, it directly activates the pathway via local mechanical load. These two forces act together to reverse the obesity-induced inhibition of the Wnt/β-catenin signal, ultimately leading to favorable outcomes including enhanced osteoblast activity and suppressed osteoclast formation, thereby improving bone metabolic homeostasis. This mechanism not only deepens our understanding of the role of exercise in protecting bones, but also provides a solid theoretical basis for using exercise as a non-pharmacological strategy to treat obesity-related.

This study conducted an endpoint assessment immediately after the completion of the 10-week intervention, which allowed us to demonstrate that exercise ultimately promotes bone formation and inhibits bone resorption. However, bone remodeling is a dynamic, multi-stage continuous process. It is well-known that mechanical loading stimulation on bones first triggers a transient activation of bone resorption, followed by sustained bone formation, and there may be an anabolic window period throughout this process. Due to the lack of dynamic observations at intermediate time points during the intervention (e.g., Week 2, Week 4, Week 6) in this study, we were unable to depict detailed kinetic curves of exercise-induced changes in bone metabolism markers (such as P1NP and CTX-1) or the expression of key factors in the Wnt pathway. Therefore, we cannot determine at which specific stage of the intervention exercise exerts its strongest effect, nor can we completely rule out the possibility of a transient increase in bone resorption that may occur in the early stage. Future studies, by performing longitudinal analyses at multiple time points, will be able to more accurately reveal the temporal mechanism of aerobic exercise against obesity-related bone loss and provide key data for optimizing the duration and frequency of exercise interventions, and future studies that use CT to assess the dynamic changes of adipose tissue will be of great value for comprehensively interpreting the interaction between lipid metabolism and bone health during exercise response.

This study demonstrated the connection between obesity and bone turnover. Obesity induced by HFD leads to decreased bone formation, increased bone resorption, and increased adipogenesis. Aerobic exercise, however, mitigates changes in bone structure. Our findings suggest that the impaired bone mass accumulation caused by obesity may be due to the inhibition of the WNT signaling pathway, and exercise intervention may restore its activity. The results provide a foundation for further exploration of the mechanisms of obesity-induced impaired bone mass accumulation. However, the mitigating effect of exercise on obesity-induced bone loss was not immediate. Therefore, in subsequent studies, different forms of exercise will be investigated, focusing on enhancing the WNT signaling pathway as an effective intervention to prevent or improve bone loss in obese patients.

## Mini-abstract

This study reveals that 10 weeks of aerobic exercise improves the imbalance of glycolipid metabolism and bone metabolism in high-fat obese male mice by activating the WNT pathway and regulating adipokines, inhibits bone resorption and promotes bone formation, providing a basis for exercise intervention for obese-induced bone loss.

## Data Availability

The raw data supporting the conclusions of this article will be made available by the authors, without undue reservation.
