# Supplementary figures and images for: Effects of aerobic treadmill exercise on the bone turnover in obese male mice
Source: Front Cell Dev Biol. 2025 Nov 6;13:1650496. doi: 10.3389/fcell.2025.1650496 (PMC12631450; doi:10.3389/fcell.2025.1650496)

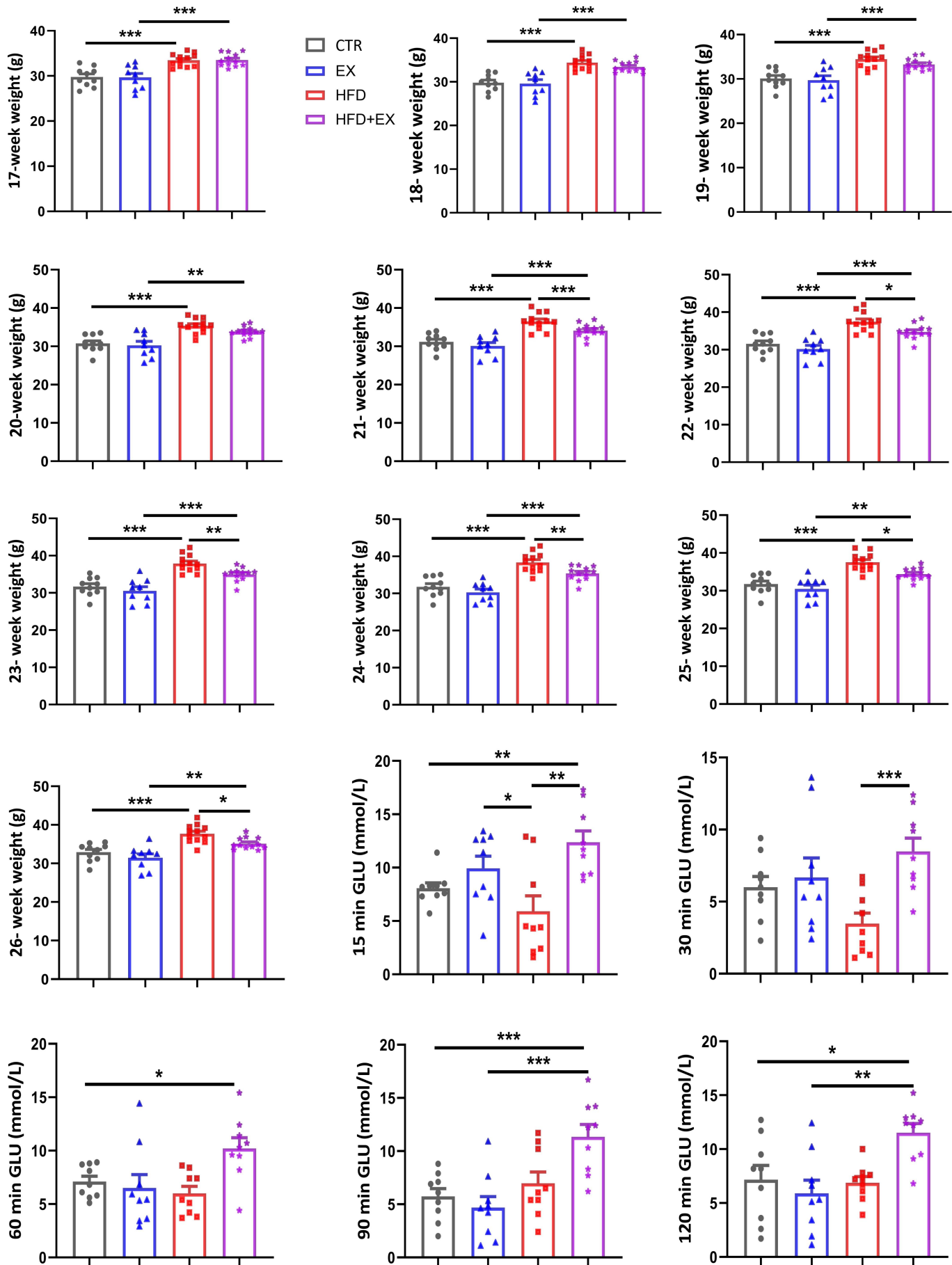

Supplement: Supplementary file 1 [file DataSheet1.pdf]
